# Supplementary material for: Are carbon emissions trading and green financial instruments synergistic? -Comprehensive quantitative research based on content analysis
Source: PLoS One. 2024 Mar 7;19(3):e0298601. doi: 10.1371/journal.pone.0298601 (PMC10919601; doi:10.1371/journal.pone.0298601)
Supplement: S1 Data — (ZIP) [file pone.0298601.s001.zip › Data/地级市Z_F工作报告.zip!/╔┬╬≈/░▓┐╡╩╨/└┤╫╘╣1⁄2╓┌║┼í╛┬φ┐╦╩2╛▌═°í┐_2015.docx]

各位代表:

　　我代表市人民政府向大会作工作报告，请予审议，并请政协委员和列席会议人员提出意见。

　　一、2014年工作回顾

　　在市委的坚强领导和市人大、市政协的监督支持下，全市上下共同努力，发展稳中有进，社会和谐稳定。去年完成生产总值689亿元，增长11.7%；财政总收入66.5亿元，增长13.3%，一般预算收入28亿元，增长13%；城镇居民人均可支配收入2.5万元，农村居民人均纯收入7490元，分别增长11%和13%；单位生产总值能耗下降4.5%，较好地完成了市三届人大五次会议确定的目标任务。

　　（一）项目推进有力，投资拉动明显。全社会固定资产投资605亿元，增长25.5％，民间投资增长26.9%，占投资总量的61.6%。西北（国际）天贸物流城、金属镍、钒氮合金、帝奥电梯等产业项目，安平高速、阳安铁路复线、西成客专（宁陕段）、石紫岚（G541）、平镇公路、东郊变电站等基础项目，汉江综合整治、引汉济渭、重点防洪工程、水保流域和瀛湖湖泊治理等生态项目扎实推进。安康新机场可研通过批复，高客站、紫阳和白河3座汉江大桥、330千伏变电站等建成。招商引资实际到位资金320亿元，增长23%。

　　（二）园区发展迅速，循环产业壮大。“三区两园”和“飞地经济”园区综合承载力提升，成为经济重要增长极。新型工业化进程加快，规模以上工业增加值增长18.3%（增速全省第二），新增规模工业企业72户，非公有制经济占比52.9%。现代农业加快发展，粮食产量86万吨，院地合作深入推进，生猪、茶叶、魔芋、核桃、山林经济等基地、龙头、品牌、质量有了长足进步。生态旅游发展水平提升，各板块依托自身优势纷纷发力，重点景区建设加快，与陕文投合作完成瀛湖旅游产业基地规划和公司组建，新增2个国家4A级景区，接待游客、旅游综合收入分别增长16%和22%。带动服务业发展，新增限额以上商贸企业45户、劳动密集型企业53户，社会消费品零售总额增长12.5%。

　　（三）突破重点难题，城镇化步伐加快。以中心城市为龙头，移民搬迁和保障性住房建设为抓手，城镇化率达42.8%。着力破解体制机制问题，集中精力解决中心城市18类67个难点问题，完成市政投资37亿元。龙舟文化园、江滩公园、东坝防洪、永安门、滨江大道三期、安运司和供销车站搬迁、一桥加固及交通组织、3座人行天桥建成，创建国家卫生城市通过综合评审，启动国家森林城市创建；针对“上学难”和“出行难”问题，推动教育、医疗卫生资源优化配置和公共交通体制改革，实施城区“五校北迁”和12所学校改扩建；突出公共交通社会性、公益性，收回公交线路经营权，组建市公共交通有限责任公司和两家公车公营出租车公司，更新投放新能源公交车114辆、出租车130辆。加快月河川道城镇带、石紫岚沿江生态经济带以及县城、重点镇和新型社区建设。避灾扶贫搬迁2.2万户8.36万人；保障性安居工程开工3万套，建成2.25万套，累计竣工入住7.4万套。农村居民进城入镇8.7万人。

　　（四）重点改革深化，发展活力增强。政府机构、镇村综合改革、事业单位分类改革按要求稳步推进，建成市级政务中心，取消下放200项行政审批事项，有效激活了市场主体，新增企业近3000户，同比增长77.6%。教育、文化、医药卫生、城镇社区管理、公安国土体制改革等取得实效。汉滨公共卫生服务责任医师团队模式、白河“秦楚医疗联合体”等做法，受到上级肯定和群众拥护。以农村土地、农村金融制度和新型农业经营体系创新为重点的农村改革不断深化。旬阳“民生金融创新”、石泉“金融精准扶贫”以及月河川道“新型农业经营主体金融培育示范带”创建工作稳步推进；财政金融创新有力拉动了社会资本向要素市场聚集，财信担保能力达到76亿元，发展小额贷款、担保公司、典当行36家；金融机构各项存款、贷款余额分别增长12%和23.8%，存贷比53.6%（居陕南第一位）。（五）投入力度加大，民生持续改善。按照补短、兜底的要求，完成民生支出165亿元，同比增长36.4%。集中办好“十件实事”，新增城镇就业1.6万人，农村劳动力转移就业64万人。城乡社会养老、城镇基本医疗、工伤、生育保险保障水平提高。教育项目完成投资7.3亿元，提前完成学前教育三年行动计划，安康职业技术学院新校区（一期）建成，石泉、平利、宁陕创建为国家义务教育发展均衡县。加强公共卫生和医疗服务体系建设，整合城区卫生资源，新建或改扩建县级医疗服务机构7个、镇卫生院69个、村卫生室640所，完成卫生投资2.7亿元。完善计生贫困家庭、失独家庭帮扶机制，稳定低生育水平。全面加强生态建设特别是汉江水质保护和大气污染防治，实施十项治理行动，造林绿化66.3万亩，环境质量持续改善。科技创新能力增强，10项科技成果获省政府表彰。文化产业增加值增长10.6%，博物馆、西城阁、高新儿童乐园一期建成。完成体育健身工程277个，580处校园体育设施对群众开放。完成专项扶贫投入27亿元，实现10.56万贫困人口脱贫。建成4所区域敬老院、8个县区老年公寓、20个社区日间照料中心和100个“农村幸福院”。创新社会治理，信访维稳、安全生产工作加强，公众安全感居全省第二。

　　（六）作风持续改进，行政效能提高。深入开展党的群众路线教育实践活动，严格执行中央八项规定，坚决整治“四风”；开展“小金库”、招投标、国有产权交易、政府采购等专项治理，“三公”经费下降38%。全市行政监察机关查处违反政纪案件252件348人。组织33个部门对255件重点任务和民生实事向群众公开承诺，跟踪督办，问责问效，担当意识增强，落实力度加大。自觉接受市人大及其常委会的法律监督、工作监督和市政协的民主监督，办理人大代表议案和建议229件、政协提案278件。80名人大代表、政协委员和群众代表旁听市政府常务会、100多名企业代表参加政府全体会、285名公民代表参加“走进市政府”活动、100名在校大学生到机关见习。审计监督力度加大，廉政建设加强，行政效能提升，成功创建为全省依法行政示范市。其他各项工作也取得了新成绩。

　　2014年，在宏观环境复杂、经济下行压力大的情况下，经济增速仍高出全省2个百分点，主要指标增幅居全省前列（一产、二产增速、非公有制经济占比全省第一，生产总值、工业增速全省第二，投资增速全省第四），发展质量效益进一步提高，公安工作、精准扶贫、避灾扶贫搬迁、保障性住房、医药卫生体制改革、金融改革创新、审计全覆盖等工作受到上级肯定。成绩来之不易，这是市委统揽全局、科学决策的结果，是市人大、市政协大力支持、有效监督的结果，是全市人民同心协力、艰苦奋斗的结果。我代表市人民政府，向广大干部群众，向人大代表、政协委员和各民主党派、工商联、人民团体和社会各界人士，向离退休老干部、驻安解放军、武警和消防官兵、政法干警，向关心支持安康发展的社会各界，致以崇高的敬意和衷心的感谢！

　　在肯定成绩的同时，我们也清醒地看到发展中面临的突出问题和深层次矛盾：发展不足，群众生活水平不高，脱贫致富和全面小康任务艰巨；产业支撑和自主创新能力不强，经济增长缺乏内生动力；资源环境约束趋紧，重大项目推进不够有力；发展环境不优，政府职能转变、改进政风行风还需持续用力。对此，我们将直面问题、勇于担当，采取有力措施加以解决。

　二、2015年目标任务和措施

　　2015年全面深化改革、全面实现年度和“十二五”规划目标，面临各种困难和挑战，任务艰巨而繁重，特别在严格的环境和资源约束下，转方式、调结构更加紧迫。经济新常态既是我们爬坡攻坚的关键期，也是奋力赶超的机遇期。我们要靠主动作为、实干创新适应新常态，把质量效益作为优先选项，把持续增长作为根本保障，把生态和民生改善作为追求目标，“蹄疾而步稳”地推动各项工作。

　　政府工作的总体要求是：全面贯彻落实党的十八大和十八届三中、四中全会以及省委、市委全会精神，坚持生态立市，坚定循环发展，稳中求进，提质增效，着力改善基础条件，壮大循环产业，深化重点改革，推进新型城镇化，保障和改善民生，确保经济健康快速发展、社会和谐稳定。

　　发展的主要预期目标是：生产总值增长11.5%，财政总收入和一般预算收入分别增长8%和12%，城乡居民收入分别增长11%和12%，人口自然增长率控制在5‰以内，汉江出境断面水质保持国家Ⅱ类标准，全面完成“十二五”规划目标。

　　围绕上述目标，重点做好八个方面工作。

　　（一）抓住关键支撑，确保经济平稳快速增长

　　把稳增长放在经济工作的首位，支持实体经济，进一步激活民间资本，发挥投资、消费的拉动和企业的主体作用，确保增长有速度、有质量、有效益。

　　保持投资增长。实施200个市级重点项目，确保全社会固定资产投资增长23%。围绕“十三五”规划编制，加强全局性、基础性、战略性项目谋划，力促一批项目进入中省规划。围绕循环产业发展，实施116个产业类项目，完成年度投资83亿元；推进安康火电厂、白河、旬阳水电站、旬阳烟厂技改开工建设，建成天贸物流城一期、金属镍、泸康白酒扩建、南水汽配等。围绕基础条件改善，加快重大交通项目以及引汉济渭、月河补水、重点水源工程、洞河水库等水利项目，汉江综合整治、瀛湖生态环境保护、中小河流治理、“两厂”建设等生态项目建设；加强供电电网建设，开工酒泉至湖南特高压直流输电工程，完成基础项目投资110亿元。加大棚户区改造、保障性住房、社会事业、养老服务等民生项目建设力度，完成民生项目投资78亿元。激发民间投资活力，推动社会资本进入重点产业、基础设施和公用事业等更多领域。

　　壮大实体经济。坚持抓大不放小，优化发展环境，发展民营经济，增强市场和企业信心。加大“四上”企业培育力度，特别是产值过5亿元企业和科技创新、成长型的中小企业、劳动密集型企业，新增125户“四上”企业。建设中小企业创业和孵化基地，推动全民创业、大众创业、青年创业。把扩大产品销售作为长期战略，帮助企业创新营销模式，推进特色产品进北京、上海、广东、天津、西安等市场；深化金融创新，发挥财信担保、金融机构的作用，针对性解决企业发展中面临的资金难题。实施部门包联工业企业制度，对各类经济组织一视同仁，精心培育和扶持，确保工业增长18%以上。

　　激活消费需求。把增加城乡居民收入作为一项根本任务，保持物价基本稳定，提升居民消费能力和预期。瞄准群众多样化需求创新消费业态，大力发展旅游、文化、休闲、体育、家政、养老和健康医疗服务等新兴消费；加快光纤宽带网络、第四代移动通信网络等信息基础设施建设，发展电子商务和信息消费，力争城区“一江两岸”及重点景区免费Wi-Fi全覆盖；加快26个重点文化项目建设，促进文化产业发展。实行公租房与廉租房、经济适用房与限价商品房并轨，探索收购商品房补充保障房路子，放宽公积金支付房租条件，促进房地产业健康发展。加强农村流通体系建设，推动连锁经营、物流配送等向农村拓展，挖掘农村居民消费潜力。扶持重点外贸出口企业，加快安康二类口岸和海关出入境检验检疫机构建设，提高经济外向度。（二）推进产业升级，加快构建循环产业体系

　　在加快发展中推动转型升级，以生态友好型产业为重点，促进一、二、三产业融合发展。

　　把工业做大做强。坚持工业强市战略不动摇，加快新型工业化进程，在改造提升传统产业的同时壮大新兴工业，引导产业链向高端攀升。装备制造力促陕汽宝通、通用、神达、俊达、双环锻造、南水汽配、华旭钢构、亨通汽车排气管等达产达效，打造区域专用汽车和零部件生产基地。新型材料以金属、非金属和建筑新材料为重点，加快白银铅锌开发、有色中科纳米、金属镍产业园、钒氮合金、汉滨绢云母、江华钡盐等企业发展，努力壮大新型材料工业。发挥秦巴特色医药资源优势，壮大正大、北医大、镇坪制药等中成药生产企业，以及永宏、天源等中间体企业，推进黄姜皂素清洁生产。富硒食品以陕煤真硒水、旬阳羊山矿泉水为重点做好涉水产业文章，加快富硒茶产业发展，推进烟厂技改，开发富硒新产品，延长产业链。

　　把农业做优做特。以产业园区为载体，实施好国家级特色（富硒）高效农业院地合作示范区规划，打造富硒特色高效农业产业链。保持定力、久久为功，不朝秦暮楚，集中精力推进生猪、富硒茶、魔芋、核桃、现代渔业、山林经济发展，重点抓主产县、基地镇、专业村、大户和龙头企业，促进适度规模经营。以市场和消费者需求为导向，突出生态、富硒特色和品牌，用现代商业和营销理念，探索从田间到餐桌的安全农产品产业链，为消费者提供安全、健康、有机的特色农产品。抓好第二轮退耕还林，突出“一江两岸”、景区园区、城镇（社区）周边、公路沿线植树造林，重点建茶叶、核桃等经济林果园，发展林下种植100万亩、林下养殖1000万只（头）。在川坝、河谷地带大力发展设施蔬菜，在重点区域发展烤烟、蚕桑、绞股蓝、特色种养等，多渠道增加农民收入。推进农业生产经营方式创新，保障农产品质量安全，培育职业农民1000人。强化土地规划统筹、用途管制、用地节约和执法监管，严厉打击土地违法，严守耕地红线，稳定粮食生产，强化水利支撑，增强农业可持续发展能力。

　　把旅游做精做美。以瀛湖旅游产业基地为龙头，以核心景区和特色旅游板块为重点，促进旅游与文化、生态、城镇、产业、美丽乡村融合发展，做美山水，打造精品景区，提升大南宫山、双龙旅游度假区、后柳水乡及中坝峡谷自驾游营地、宁陕全域旅游、平利美丽乡村、汉阴凤堰古梯田和巴山百里旅游画廊等景区水平。围绕吃、住、行、游、购、娱等要素，抓好旅行社、宾馆酒店、特色餐饮、景区交通、旅游产品等，完善旅游配套服务体系；在重点风景名胜区利用历史文化资源，挖掘一个美丽的传说或故事，编排一部戏或一台特色节目，促进文化与旅游融合。面向西安、重庆、成都等强化宣传推介，加强与周边城市的深度合作，做精秦巴生态、汉水风情、美丽乡村等旅游线路，主动融入秦巴生态旅游圈，接待游客和旅游收入分别增长15%和21%。探索旅游、文化、生态“三位一体”的服务业发展模式，努力把安康建成山水休闲旅游、健康养生基地。

　　（三）坚持交通先行，强力推进区域交通枢纽建设

　　以突破瓶颈制约和提升互通互联水平为重点，努力构建现代交通运输和物流体系，增强发展的保障能力。

　　加快实现互通互联。围绕“打开通道”，建成安平高速，开工安康富强机场、平镇高速，加快西城客专（宁陕段）、阳安复线、石紫岚、平镇二级公路、大岭铺至安康东直通线、高等级标准航道建设，加大安岚高速、桐木至旬阳、石泉至大河坝高速连接线以及月河快速干线、包海客专（西康城际）、汉康城际、安张铁路、新安康火车站等前期工作力度。围绕“完善路网”，开工白河至旬阳、旬阳至安康、旬阳至小河、宁陕至两河公路升等改建工程。围绕“提升功能”，加快北环线、县城国道改线、四座汉江大桥（城东、汉滨石梯、石泉汉江、长阳）建设；开发瀛湖至紫阳、瀛湖至岚皋佐龙水上“一日游”，加快安康中心港、紫阳港客货码头迁建、火石岩码头改造以及40个农村渡口建设。

　　优先发展公共交通。坚持“方便群众、综合衔接、绿色发展、因地制宜”的原则，建设高效快捷安全的“大公共交通”体系。统筹城市公交、城际和农村客运，促进城乡公共交通一体化发展。科学规划公共交通站场和线网布局，优化与机场、火车站、高客站、港口等重要交通节点设置和换乘。加快城市综合客运枢纽、调度中心、港湾式停靠站、“一卡通”、停车场、自行车道、步行道等配套设施建设。提高公交线网密度和站点覆盖率，推进公交线路向集镇、学校、园区、景区等延伸。加强出租车规范管理，实现运力和运量的合理配置，促进出租车行业健康发展。深化“122”高速公路路警联合执勤、“125”山区道路联合管理模式，建立公共交通安全监管体系，促进公共交通安全发展。

　　推进物流产业发展。围绕建设区域物流中心，依托天贸物流城，规划建设冷链物流园、农产品物流中心等。统筹网上与网下、基地与产地、商贸与仓储，促进电子商务、物流配送和快递业发展。加快各类专业市场和“商圈”打造，建设高新重型机械、诚鹏机电、静宁市场以及秦巴明珠商贸城、泸康物流园、宝业仓储物流园等，培育壮大物流骨干企业，提高物流信息化水平。以降低流通成本为重点深化流通体制改革，促进交通运输业发展，打造立足陕南辐射秦巴的产品集散地。（四）释放园区活力，加快县域经济发展

　　坚持点上开发、面上保护，发挥比较优势，促进要素聚集，释放“三区两园”和“飞地经济”园区发展的新能量，带动县域经济发展。

　　培育经济增长极。围绕打造“投资洼地”和“产业高地”，创新“三区两园”和“飞地经济”园区管理体制、运行机制和服务体系，积极推进政府与社会资本合作PPP模式，改善基础条件。高新区在引领改革、创新机制上做表率，优化创新环境，集聚创新资源。围绕天贸物流城做好产业配套；围绕新型材料、装备制造、生物医药产业发展，加快金属镍产业园、钒氮合金、南水汽配、北医大制药、帝奥电梯等企业壮大；围绕打造“商圈”，完善商贸、餐饮、市场等要素，加快服务业发展，推进农发行、长安银行北迁和金融、保险等服务业入驻；围绕聚集“人气”，加快安康学院、职业技术学院北迁以及高新学校、公共卫生服务中心、儿童医院、妇幼保健院等公共设施建设；围绕创建国家高新区目标，加快总部经济和科技创业孵化园建设，搭建产学研结合、基地孵化、自主创新为主的创新平台，建设人才吸引高地。恒口示范区围绕城市副中心、省级重点示范镇建设，加快道路、市政、环卫等基础设施和学校、医院等公共设施，以及集中安置社区和产业园区建设，在招商上着力、培育产业上突破，促进产城融合，加快创建镇级市。瀛湖生态旅游区支持陕文投安康公司加快发展，强化服务保障，按规划选准突破口，启动景区和基础建设；进一步完善体制机制，严格镇村规划管理，改革旅游船舶、水上运输管理经营体制，规范水产养殖、农家乐、餐饮等经营行为，防止无序发展，全面提升软硬件和管理水平。县域工业集中区推动由“摊大饼”粗放式、数量型向内涵式、创新型发展转变，由初级产品加工向精深加工、产业链中高端延伸，以市场需求为导向，提高企业入园门槛，全面提升质量效益。建成标准化厂房16万平方米、引进千万元投资入园企业50户。“飞地经济”园区进一步强化市级统筹、县区主体、部门服务，加大基础建设和招商引资力度，加快企业和项目落地，着力培育市场主体，力促“飞地经济”成为发展的新引擎。现代农业园区完善基础配套，抓住重点龙头企业，发挥带动作用；着眼销售、瞄准市场，抓标准化、规模化生产，大力发展电子商务、农超对接等新兴业态，提升农产品质量、品牌和营销水平。

　　加快县域经济发展。坚持分类指导、重点扶持，彰显特色、错位发展，改革有关支持配套政策，建立支持县域园区及产业发展的长效机制，放大县区比较优势，加强产业配套协作，构建特色鲜明、协调互动、充满活力的县域经济发展格局。统筹抓好县城及副中心、工业集中区、新农村建设以及民营经济发展、避灾扶贫搬迁，支持汉滨区打造百亿产值工业集中区和服务业快速发展，发挥龙头引领作用；以撤县建市为目标，支持旬阳县创建省级高新区，加快改革发展步伐；支持平利县建设国家级现代农业园区；支持其他县发挥比较优势，壮大特色产业；支持县区自主探索改革和工作创新。抓住国道541建设的机遇，突出规划引领、移民搬迁、产业支撑和特色小镇、美丽乡村建设，努力把石紫岚沿江生态经济带打造成绿色经济走廊。

　　（五）统筹城乡发展，有序推进新型城镇化

　　把建设“秦巴明珠”作为长期战略，围绕让城乡更加宜居、环境更加优美、“明珠”更加璀璨，坚持以人为本、产城融合，突出中心城市辐射带动作用，推动月河川道城镇带及县城、重点镇（社区）协调发展，构建和谐宜居、生态优美、特色鲜明、充满活力的城乡一体化发展格局。

　　建设优美宜居的区域中心。以创建国家森林城市和省级文明城市为抓手，全力推进“突出核心、提升江南、重心北移”战略，突出重点、突破难点，完善城镇功能，增强在秦巴区域的辐射力和带动力。围绕建设宜居宜业宜游的生态城市，不求大而重在美、不求洋而重在“特”、不求奢华而重在“宜人”。坚持“湖城一体”打造“一江两岸”城市核心，构筑以汉江为轴线的点、线、面结合的景观格局，推进“一江两岸环江50里”建设；抓住棚户区改造政策机遇，重点实施东关（静宁市场及南正街改造）、西南（西郊公园建设、棚户区及瀛湖路改造、片区开发）、东南（高井二期、南山路、高井一路及安置大社区建设）、东西坝片区（基础设施建设及棚户区）改造，提升市民幸福指数。围绕建设区域中心，进一步拓展城市发展和承载空间，加快城东和石梯大桥、北环线、长春路、机场快速干线等重心北移的控制性工程建设，同步规划和整合江北学校、幼儿园、医院、文化、体育等设施，加快高新区、火车站片区、月河生态城建设。

　　统筹县、镇（社区）和新农村建设。按“城乡一体、产城互动”的要求做优、做精、做美县城，在彰显特色上着力，突出市政和公共服务设施建设，围绕整洁、畅通、增绿、治违以及出口整治等抓提升，让精细管理全覆盖，让县城（集镇）处处见绿、身边处处见景。以12个县域副中心、4个文化旅游名镇为重点，培育以工业主导、商贸带动、现代农业或旅游休闲为特色的优美小镇。加强农村新型社区产业配套和公共设施建设，实施“十镇百村”美丽乡村创建，提升村庄规划水平，统筹抓好农村基础设施、公共服务、垃圾收集、污水治理、造林绿化、安全饮水等建设，加快村级公益事业发展。

　　提升城镇内涵和综合承载力。城镇建设从以基础设施带动转到更加注重社会建设和公共服务配套上，加快推进城镇路、管、电网、环卫、绿化、停车场、各类市场以及教育、医疗、文体、休闲、健身等公共设施建设。强化城镇规划管理，指导各县制定修编相关规划，全面启动建制镇规划编制工作，在“三区”和石泉开展“多规合一”试点；提高城镇土地利用率，严控增量，盘活存量，节约集约用地。创新投融资机制，鼓励社会资本参与城镇公用设施投资运营。围绕建设智慧城市，促进跨部门、跨行业的政务信息共享和业务协同，完善城市交通通信、社会治安、市容环境等智能化信息系统。加快推进农村居民市民化，进一步健全社保、医疗、教育、保障性住房等社会保障跟进机制，让所有进城的农村居民平等享受市民待遇。　（六）深化改革开放，以创新驱动发展

　　改革是经济发展和社会进步的永恒动力。我们要靠改革激发新活力，靠开放释放新推力，最大程度地激活市场主体、增强发展活力。

　　深化重点领域改革。以解决重点问题为突破、以维护群众利益为导向，积极推进体制机制创新。在转变政府职能方面，进一步理顺政府与市场的关系，简政放权，减少审批，减少环节，逐步实现由事前审批向事中、事后监管转变。建立部门权力清单，承接好省、市下放的权限，提高效能、优质服务。完成政府机构、镇村综合改革，加快事业单位分类改革，推进行业协会、商会与行政机关脱钩，建立健全政府购买公共服务机制。实施商事制度改革，允许“一照多址”和“一址多照”，试行“三证合一”，进一步放宽市场主体登记条件。加大对政府管理资源的监督力度，建设工程招投标、土地招拍挂、政府采购等有形市场，营造公开、公平、公正的市场秩序。在经济领域改革方面，围绕发挥市场在资源配置中的决定性作用，着力推进城镇化发展体制机制、财税金融改革创新、公共资产管理、发展非公有制经济、公共资源配置、诚信体系建设、园区体制机制、国资监管体制等改革，创造条件组建交通、文化投资发展公司。以实施全省农村改革试点为契机，增强农村经济社会发展活力。在社会领域改革方面，重点推进医药卫生、教育、文化、科技、生态环境、创业就业、社会保障等方面的改革。

　　强力推进对外开放。今天的安康，大开放的使命比任何时候都更加紧迫。我们将加大力度推进思想解放、观念创新、方式转变、环境优化，敢于用比较优势整合吸引社会资本，敢于到外部市场聚集要素、寻求合作。提升“走出去引进来”的水平，学习先进经验，开阔视野，增强战略思维和世界眼光。把招商引资作为对外开放的第一抓手，主动融入“丝绸之路经济带”，向东向西开放，突出新兴工业、现代物流和服务业、劳动密集型企业招商，紧盯国内500强、行业领军企业以及知名央企、陕企、民企，瞄准高校、科研院所科技成果、专利技术，“一对一”精准招商、产业链招商、驻地招商、以商招商，增强全民开放、招商的活力和动力。创造更具包容性的环境，让外来资本有市场、外来企业有机遇、外来人才有事业，引得来、留得住、能发展。

　　实施创新驱动。把创新作为转方式、调结构、奋力赶超的关键，建立健全以企业为主体、市场为导向、产学研用相结合的创新体系。加大力度推进科技体制改革，多渠道增加科技投入，创新人才引进培养使用机制，实施“百名硕博进安康”、“千名大学毕业生创业”、“培育万名能工巧匠”工程。强化企业技术创新主体地位，支持企业建立技术研发平台，鼓励产品、品牌、产业组织和商业模式创新。深化与中国农科院、药科大学、西安交大、陕西师大、安康学院等合作。组织市级科研院所围绕市场需求、产业发展需要，坚持自主研发和引进推广应用并重，加速科技成果转化。让一切创新创业的源泉充分涌现，让创新创业的“星星之火”成燎原之势，推动经济从投资驱动向创新驱动迈进。

　　（七）强化生态建设，加大环境保护力度

　　把生态作为安康可持续发展的关键，深入推进国家主体功能区建设试点示范，加快形成符合生态文明要求的生产生活方式和消费模式。

　　加强生态环境建设。科学划定生态保护红线，抓好第二轮退耕还林、国家森林城市创建，造林绿化50万亩；加强山水林田湖的保护和修复，加大工业点源和农村面源污染治理力度，抓好土壤、地下水、重金属污染防治，深化农村环境综合整治。依法严厉惩治环境违法行为，把整治乱批乱建、违法采石开矿向山区深处延伸。强力推进节能减排降耗，全面完成“十二五”主要污染物减排目标。探索建立环境保护多元化投资、建设、管理和运营模式，倡导和推广绿色消费、低碳出行、文明健康的生活方式，让广大群众积极参与、共建共享。

　　保护好汉江母亲河。围绕“源头严防、过程严管、后果严惩”，建立汉江水质保护联席会议、目标考核和责任追究制度，全面推行“河长”制，实施网格化、流程化监管，形成汉江水质保护的长效机制。在完善县区“两厂”配套管网和规范运行的基础上，用两年左右时间基本建成沿江重点镇（社区）污水垃圾处理设施。完善汉江水质实时监控体系，建成南水北调汉江水质保护应急指挥中心，保护好我们赖以生存的母亲河。

　　开展“治污防霾、保卫蓝天”行动。雾霾的警报已经拉响，我们决不能掉以轻心，必须转观念、转生产生活方式，打好治污防霾的全局战。抓住“压煤、抑尘、控车、禁燃”等关键环节严控大气污染源，加快“气化安康”步伐，大幅提高工业、交通以及民用天然气的普及率，实施重污染企业技改和燃煤小锅炉淘汰，大力推进“煤改气”、“煤改电”和“油改气”；强化对工地和道路扬尘的监管，加大机动车尾气污染治理力度，加速淘汰黄标车，提高城镇主要道路机扫率，严禁秸秆焚烧，抓好农村清洁能源应用，实施PM2．5监测和信息发布。全社会共同努力，确保空气质量全国全省领先，决不以牺牲生态环境这个最宝贵的资源和最大的优势为代价换取经济增长，决不让雾霾遮住安康美丽的天空。（八）保障改善民生，为群众多办实事

　　把有限的财力用在办好群众最关注、最迫切、最广泛的实事上，让群众更多享受改革发展的成果。

　　办好十件实事。一是推动创业就业。加大创业孵化、技能培训、就业援助力度，统筹促进高校毕业生、农村转移劳动力、退役军人、返乡农民工、困难群体等就业，建成大学生创业孵化园，支持50家中小企业和2000人创业，发放小额担保贷款4亿元；围绕产业发展以及公交、社区服务等公共事业需求，免费技能培训2万人；转移就业农村劳动力60万人，新增城镇就业1.5万人，完成农民实用技术培训10万人次。整合各类扶助资金，不让一个考上大学的孩子因家庭困难上不了学。二是提升办学能力。完成安康职业技术学院新校区建设和“五校北迁”以及安康中学、阳光学校、汉滨初中、兴安初中改扩建，新建东坝、江北、黄沟小学并按期投用，原汉滨初中改办小学实现秋季招生。实施中心城区江北片区教育资源整合。三是改善医疗条件。整合市中心血站、疾控中心、安康疗养院和120指挥中心北迁，开工建设市级公共卫生服务中心，建成市妇幼保健院。实施市中心医院、中医院、人民医院综合改造提升，加强重点专科建设，突出特色、错位发展。把基层基础建设的重点放在县城和区域医院建设上，提升医疗水平和服务能力。整合卫生计生公共资源，新改建县、镇村、社区卫生服务设施340个。四是实施市政民生工程。城东大桥主体完工，实施“三桥一路”提升，改造金州路、兴安路、316国道江北安旬路口至高新区段以及城区交通堵点，建成6座人行天桥，新增500个泊车位，完成殡仪馆搬迁，建成江滩公园二期以及兴安、静宁、诚鹏等市场。进一步优化配置市区两级公共资源，下决心腾退部分公共资源用于群众休闲、锻炼、停车场等公共服务。推进背街小巷、城中村、城乡结合部无物业管理小区清扫保洁常态化，逐步配套公共服务设施。五是发展公共交通。实施平利、石泉公交客运城乡一体化试点，安康高客站二期竣工，建成江南、高新公交枢纽和21个港湾式停靠站，实现中心城市规划区公交全覆盖，并逐步向郊区延伸，开通城区至恒口、关庙等公交，新增公交车50辆，更新出租车100辆，提升市民公交出行的便捷性、舒适感和满意度。支持有条件的县城公交逐步向集镇延伸。六是提高天然气入户率。下大力解决天然气入户的“最后一公里”问题，建成城区2座加气站，加大城市管网、老旧小区改造接入工作力度，中心城区7万户25万人用上天然气。加快各县城门站和加气站建设。七是实施文体惠民工程。实施中心城市“安康文化大本营”工程，通过国家公共文化服务体系示范项目创建验收。建成市体育中心综合训练馆、“藏一角”博物馆，开工　　建设汉江大剧院、香溪书院、江北体育场馆，安康博物馆对市民开放。实施418个社区公共体育工程。创造条件将“一江两岸”公共设施全部用于文体事业。八是提升住房保障水平。推动建筑业转型升级，开工保障性住房2.78万套，建成2.3万套，新增发放租赁补贴2800户，创建“和谐社区、幸福家园”示范小区，加强保障性住房规范管理。围绕“提速、提效、规范”，推进精准搬迁、精细管理、精确施策，建设避灾扶贫搬迁集中安置社区290个，完成2.5万户9.5万人搬迁安置。九是实施健康养老工程。把健康养老作为新兴产业培育，创新思路，引进民间资本建设面向西安和全市的生态休闲养老基地，在汉滨、石泉、宁陕探索发展健康养老新模式，开工建设市级生态休闲养老中心二期，建成15个标准化社区日间照料中心和100个“农村幸福院”。十是保障饮水安全。完善中心城区供水体制，逐步取消城区地下取水，保障群众用水安全。以县城第二水源（水厂）建设和解决移民搬迁安置社区群众饮水问题为重点，按照满足水量、提升水质、强化管理的要求，建成9县城第二水源（水厂），解决20万农村人口饮水安全问题。

　　发展社会事业。坚持教育优先发展，实施48个中小学、幼儿园建设和141个农村初中校舍、普通高中、农村义务教育薄弱学校改造，探索学区制、校长教师管理制度、职业教育资源整合等改革，实施第二轮学前教育三年行动计划，鼓励和规范社会力量办学。深化医药卫生体制改革，探索组建医疗联合体和分级诊疗模式，完善新农合、基本医保、重特大疾病保障制度，发展中医药事业，鼓励社会力量办医，加大基层医疗卫生人才培养力度。创新公共文化管理体制和运行机制，加强城市社区和农村文化设施建设，促进城乡基本公共文化服务均等化；深入发掘历史文化资源，支持文化艺术精品创作，加强公共文化产品和服务供给，加大文化遗产、历史文物、革命遗址保护力度，大力发展有线电视和便民广播，广泛开展群众文化和全民阅读、健身活动。加强社会救助体系建设，提高社会保障水平。强化计划生育工作，加强农村留守儿童、妇女、老人的关爱和服务。推进殡葬改革，每个县建成殡仪馆和城市公益性公墓。大力发展老龄、残疾人、妇女儿童、红十字、慈善事业，让每一个身处困境者都能得到社会关爱和温暖。围绕“两不愁、四保障”目标，加大精准扶贫力度，权责到县、工作到村、扶贫到户、精准到人，完成扶贫专项投入25亿元，实现10万人脱贫目标。

　　创新社会治理。把做好群众工作贯穿社会治理各方面、各环节，建立健全畅通有序的诉求表达、心理干预、矛盾调处、权益保障机制，改进社会治理方式，加强社区管理服务创新，激发社会组织活力，推进信息化、网格化管理。进一步强化基层基础，做好村委会换届工作。深入推进平安创建，建成“天网工程”，完善治安防控体系，加大对违法犯罪和“黄赌毒”的打击力度。强化流动人口和特殊人群管控和服务。把信访维稳“分级负责、属地管理”和“谁主管、谁负责”落到实处，畅通信访渠道，加大依法治访力度。加强法律援助工作，完善“大调解”工作体系。以对党和人民高度负责的态度抓好安全生产，健全党政同责、一岗双责、齐抓共管的责任体系，下大力排查整治安全隐患，确保企业安全生产、群众平安生活。加强食品药品和质量监管，强化检验检测能力和信息化建设。加强防汛抗旱、防灾减灾工作，保障群众生命财产安全。

　　与此同时，我们将以2020年全面建成小康为坐标，认真编制“十三五”经济社会发展规划。做好国防动员、双拥共建、国家安全、民族宗教、优抚安置、人民防空以及统计、移民、粮食、外事、侨务、对台、史志、档案等工作，支持工会、共青团、妇联等人民团体发挥更大作用。三、加强政府自身建设

　　深入贯彻落实党的十八届四中全会精神，自觉维护宪法和法律权威，建设法治政府，创新行政管理方式，增强政府公信力和执行力。

　　全面推进依法行政。把依法行政作为政府工作最基本的要求，做敬畏法律、勇担责任的政府。各级领导干部带头做学法、尊法、守法、用法的模范，提高运用法治思维和依法办事的能力。坚决执行市人大及其常委会的决议决定，自觉接受人大法律监督、工作监督和政协民主监督；在区域发展规划变更、城乡建设规划调整、大额财政资金使用和政府举债融资等重大决策出台前，向人大及其常委会报告，进一步提高建议提案办理质量和满意率。重视网络监督和网络舆情，提高与新闻媒体打交道的能力，通过媒体监督来倾听群众意见、改进政府工作。健全科学、民主、依法决策机制,做到行政决策和行政执法阳光透明、公正公平。增强“六五”普法的针对性和实效性，提高全民法治意识，推进法治安康建设。

　　强化权力制约监督。权力必须受到监督，这是一种新常态，不能有任何侥幸和懈怠，必须始终做到心中有党、心中有民、心中有责、心中有戒，对组织、人民、社会、家庭和自己负责。严格落实党风廉政建设责任制，加强廉政风险防控，政府部门全面实行政务信息、办事公开制度，让行政权力在阳光下运行。对权力集中的部门和岗位实行分事行权、分岗设权、分级授权、定期轮岗，防止权力滥用。进一步发挥审计规范权力运行的“利器”作用，在全覆盖的基础上加强重要领域、重大项目、关键岗位的审计监督，开展经常性效能和廉政监察，重视从群众反映和审计中发现线索，加大案件查办力度。坚持厉行节约，从严控制“三公”经费支出。加强公务员队伍建设，开展“学本领、强素质”活动，提高行政效能和服务水平。

　　持续加强作风建设。巩固党的群众路线教育实践活动成果，各级政府班子和领导干部带头守纪律、讲规矩，自觉践行“三严三实”，对党忠诚、干净干事，始终以发展为重，主动作为、善谋善为、敢闯敢干、勇于担当。用严格的制度和有力的措施纠正不作为、乱作为，决不姑息迁就，确保苗头性问题有效遏制、群众反映强烈的问题及时处理。力戒华而不实、夸夸其谈，以昂扬向上的精神状态，当好抓落实的“主攻手”，对认准的事、群众拥护的事、公开承诺的事，无论问题多难、矛盾多大，都要有明知山有虎、偏向虎山行的劲头，真正成为带领人民群众战胜困难的主心骨。继续实行公开承诺制，扩大承诺范围，完善督查督办、问责问效办法，把要干的事、干到什么程度、什么时间干成，向群众承诺，为群众办事，受群众监督。出水才看两腿泥，面对矛盾困难敢于亮剑，靠智慧和能力兑现承诺、取信于民。

　　各位代表，政府工作说到底就是一个“干”字。一切机遇，只有在实干中才能把握；一切难题，只有在实干中才能破解；一切愿景，只有在实干中才能实现。循环发展、干字当头，让我们在中共安康市委的坚强领导下，把责任、担当和落实作为坚持如一的根本，说了算、定了干，决不辜负全市人民的重托，为建设美丽富裕新安康而不懈奋斗。
